# P065: Vancomycin resistant enterococci among patient in Kuala Lumpur Hospital, Malaysia: the occurence and its associated risk factors

**DOI:** 10.1186/2047-2994-2-S1-P65

**Published:** 2013-06-20

**Authors:** R Ibrahim, N Ahmad, MN Aziz

**Affiliations:** 1 Microbiology, Cyberjaya University College of Medical Sciences, Cyberjaya, Malaysia, Selangor; 2 Microbiology, Institute of Medical Research, Kuala Lumpur; 3 Microbiology, Kuala Lumpur Hospital, KL, Malaysia

## Introduction

Vancomycin-resistant enterococci (VRE) are increasing in prevalence at many institutions, especially among patients with co-morbidity conditions that associated with frequent hospitalisation. It is important to determine the risk factors for this resistance microorganism as it will guide the clinician for appropriate antimicrobial therapy and infection control measures.

## Objectives

The descriptive, cross sectional study was carried out to determine the prevalence and risk factors for vancomycin resistant enterococci in Kuala Lumpur Hospital (HKL), Malaysia.

## Methods

For 12 months period, antimicrobial susceptibility testing for enterococci species were performed using disk diffusion method. E-test for vancomycin was proceed for the isolates that exhibit resistance to vancomycin by disk diffusion method. To identify the risk factors, a questionnaire was completed for all studied patients and the data were analysed using chi square and multivariate logistic regression.

## Results

The prevalence of VRE among patients in Kuala Lumpur Hospital was 1%. In chi square analysis, vancomycin usage (p=0.000, RR, 34.615; 95% CI, 5.796-206.723) showed significance risk factor. In multivariate logistic regression analysis, prolonged hospitalization (p=0.040, RR, 80.194; 95% CI, 1.212-5304.5) and vancomycin use (p=0.009, RR, 18.376; 95% CI, 2.143-165.3) were associated with potential VRE.

## Conclusion

The findings of this study will serve as an alert to the clinicians of the emergence of infections by VRE and it will encourage the implemention of appropriate infection control measures and judicious use of vancomycin in order to prevent further rise in VRE prevalence.

## Disclosure of interest

None declared
